# Orange Is the New Green: Exploring the Restorative Capacity of Seasonal Foliage in Schoolyard Trees

**DOI:** 10.3390/ijerph13050497

**Published:** 2016-05-17

**Authors:** Eli Paddle, Jason Gilliland

**Affiliations:** Department of Geography, Social Science Centre, Western University, 1151 Richmond St., London, ON N6A 5C2, Canada; epaddle@gmail.com

**Keywords:** school, greening, trees, visualization, restoration, child, healthy

## Abstract

Urban schoolyard environments are increasingly characterized by a proliferation of hard surfaces with little if any greenery. Schoolyard “greening” initiatives are becoming increasingly popular; however, schoolyard designs often fail to realize their restorative potential. In this quasi-experimental study, a proposed schoolyard greening project was used to visualize alternative planting designs and seasonal tree foliage; these design alternatives were subsequently used as visual stimuli in a survey administered to children who will use the schoolyard to assess the perceived restorative capacity of different design features. The findings indicate that seasonal changes in tree foliage enhance the perceived restorative quality of schoolyard environments. Specifically, fall foliage colour, when compared to green foliage, is rated as being perceived to be equally restorative for children. Additionally, seasonal planting, including evergreen conifers, may enhance the restorative quality of the schoolyard especially when deciduous trees are leafless. Landscape design professionals, community-based organizations, and other decision-makers in schoolyard greening efforts should strategically consider their tree choices to maximize year-round support for healthy attention functioning in children through restoration.

## 1. Introduction

Seasonal influence on human behaviour and mood is widely recognized, but not well understood, especially in school-aged children [1,2]. Among the most frequent symptoms reported as part of seasonal mood disorders among children are difficulties concentrating, irritability, fatigue, decreased activity, social withdrawal, and school problems [1].

The strategic and targeted design of children’s schoolyard environments offers great potential impact upon children’s mental and physical health and well-being, as this is an environment to which children have regular and prolonged daily exposure, and which may benefit their mental health, concentration, and ability to learn. This present work explores the influence of seasonal changes in canopy tree foliage and seasonal planting design strategies upon perceived attention restoration in elementary school children in a case study school in London, Ontario, Canada. Using a proposed schoolyard greening design as the base for the development of a three-dimensional digital visualization model, variations in planting design and seasonal foliation changes were created for use as stimulus images in a perceived attention restoration survey.

**Figure 1:**
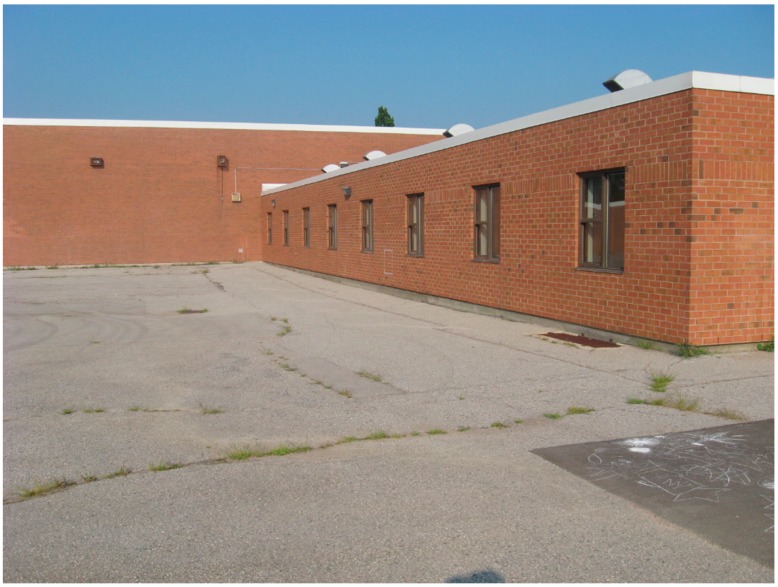
Photograph of a typical asphalt schoolyard.

Many North American schoolyards are lacking in vegetation and are predominantly surfaced in a hardscape material, most commonly, asphalt (see Figure 1).

A large expanse of forgiving turf with shade trees is a less common schoolyard experience for elementary school children. Furthermore, many schools are now removing traditional play equipment and replacing it with more asphalt, making these environments even less appealing and functional for the child user. Leading environmental designers have acknowledged this condition and are spearheading efforts to provide children with more green or natural outdoor environments that can support healthy play and learning [3]. These efforts focus on the redesign of schoolyard spaces, specifically through greening strategies. Schoolyard greening has become a niche area for landscape design professionals and organizations catering to this practice, such as REAL School Gardens, or Toyota Evergreen, have emerged.

Schoolyard greening efforts, although governed by site conditions to a certain extent, typically involve the introduction of green or natural elements, usually in the form of young native deciduous trees. In addition to simply greening the space, trees are used for a number of other desirable outcomes. Beyond the provision of shade, trees are thought to reduce extreme heat, provide clean air [4] and offer other ecosystem benefits, such as increased levels of physical activity [5]; greater social cohesion and sense of belonging [5,6]; better self-esteem, improved mood, general perceptions of health and wellness [7]; and overall improved sense of social health [8].

Another important benefit that trees provide is the potential provision of restoration. Restoration can be defined as the process of recharging depleted cognitive function and capability, which are negatively affected by prolonged directed activities or exposure to stress that produce mental fatigue [9,10]. Research on restorative environments to date has demonstrated that there is a marked effect from green domestic exposures on stress reduction, well-being and attention capacity [11,12]. Recent research on the influence of redesigning schoolyard environments in Australia has shown that such interventions can reduce stress and improve psychological well-being through attention restoration [13]. It has yet to be determined whether exposure to those natural elements and environments that are not green, such as fall leaf colours, are more or less restorative compared to purely green conditions [9,10,14].

**Figure 2:**
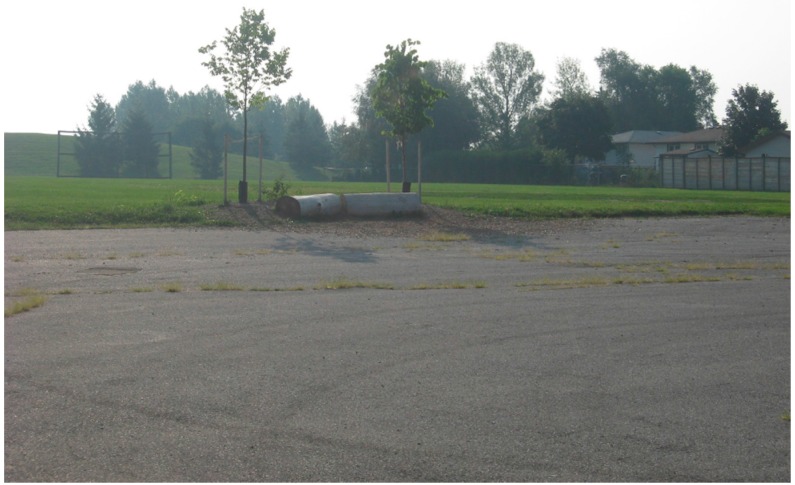
Photograph of typical schoolyard greening intervention in mid-summer.

Despite the best efforts of school yard greening initiatives, the maximum benefits of natural environments may go unrealized if tree selection focuses strictly on those that produce green foliage, as for the majority of the school year in Canadian cities, the deciduous trees either have no foliage or foliage that is not green*.* London, Ontario, Canada, which is located at the northern extent of the Carolinian zone in North America with a longitude of 42.9837°N and a latitude of 81.2497°W, has four distinct seasons wherein the majority of the trees are deciduous. The trees typically specified in schoolyard greening projects are predominantly native deciduous shade tree species (see Figure 2).

In the spring and summer seasons, the colour of the foliage on these trees is typically green. While there are many colourful flowering ornamentals that are spectacular in the spring, they are typically predecessors of fruit, which is seen as problematic in schoolyards (in the minds of administrators and maintenance staff), therefore, ornamental trees are not often used in schoolyard greening projects.

While there have been attempts to implement more innovative planting schemes that may include edible plants including fruit trees, these designs are often difficult to implement. In the Carolinian zone, in which our case study is situated, deciduous trees are typically not just green in the experience of the child user during the school year. This study will specifically address the following questions relating to the restorative quality of seasonal changes in schoolyard tree foliage.

*(1)*  How do seasonal changes in deciduous tree foliage impact children’s perception of the restorative value of schoolyard trees?
*(2)*  Does the addition of evergreen coniferous trees extend the restorative effect of schoolyard plantings during times when deciduous trees have no foliage?

### Context

#### Seasonal Mood and Behaviour Changes in Children

A well-established and growing body of research suggests that exposure to natural environments is of great importance to mental health in adults [9,13,14]. These environments are referred to as “restorative environments” and are believed to restore physical and mental health, reduce stress, improve consciousness, as well as heighten focus and attention in human subjects as outlined in “attention restoration” and “psycho-evolutionary” theories [9,14]. Research reveals faster attention recovery, higher levels of attentiveness, reductions in post-operative stress and quicker recovery for those exposed to natural scenes *versus* those who were not [15,16]. This exposure to natural settings does not have to be a physical experience; it can be in the form of views from a window or even exposure to images of natural scenes [16,17,18].

In contrast to urban scenes, natural scenes appear to provide a much greater level of attention restoration [19,20,21]. A comparative study of post-secondary students with natural views outside their dormitory windows with those that did not indicated that the students with natural views showed stronger attention capacity [19]. Even in the extreme conditions experienced in jail, prison inmates with natural views from their prison cell windows made fewer visits to the infirmary than those without natural views [22].

Subtle green exposures, such as the presence of a small number of plants on the floor of a school class room, have been shown to improve levels of perceived health and comfort by occupants and to reduce both school time missed due to illness and negative behavioural episodes [23]. It has even been suggested that consumer exposure to virtual representations of nature in product advertising may have emotional benefits that are analogous to those experienced when in contact with “real” nature [24].

A significant body of environment and behavior research has demonstrated that these benefits are also applicable to children, perhaps even to a greater degree than for adults because their attention capabilities are still developing. Faber Taylor, Kuo and Sullivan’s study [25] of children with Attention Deficit Disorder (ADD) found that exposure to natural environments lessened the severity of a child’s attention problems, and some parents found it effective to expose their children to natural environments prior to sending them into the learning environment. In studying the home environment’s restorative capacity, Wells [11] found that there was a marked improvement in children’s cognitive functioning when they moved from a poor quality natural environment to better, more restorative natural surroundings. The experience of natural environments during the school day would seem to be an even more important consideration for elementary students, since they are required to sustain prolonged effortful attention as they learn in an environment that is often full of distractions, while having less control than an adult over their attention capabilities [26,27]. Outdoor recess breaks could provide similar natural exposures in support of attention capacity or stress reduction, provided the landscape had supportive characteristics; in most North American schools, recesses and lunch break provide approximately an hour of outdoor play each day that could provide children the opportunity to recover from stress and recharge their attention capacity.

As the investigation of restorative environments for young learners narrows in scope, the focus is shifting to the role or importance of specific restorative elements. While previous research has focused on green environments for young learners in general, landscape architectural research by Mastuoka [28] has added further support for the suggestion that trees and shrubs may in fact be the most important natural feature within those landscapes. The large flat expanses of turf common in many schoolyards do not provide the same psychological or performance benefits as treed environments, nor are they preferred as much as treed environments [19,28,29,30]. For most children, their typical daily routine includes at least some exposure to green space and in the case of most of these environments, the dominant natural or “green” feature is trees. Trees, in addition to being a physically dominant feature, may have additional significance according to Smardon [31]: “They are a visible symbol of the natural world. Trees are the primary and sometimes, the last representatives of nature in the city and thus, individuals or groups may see trees as anchors of stability in the urban scene” (p. 94). Schoolyard greening initiatives featuring tree plantings which reintroduce these green “anchors of stability” coupled with engaging, practical learning about the natural world, have produced improved academic performance in children across the entire curriculum [32,33,34]. The focus of current research has become identifying which natural environments are restorative and how their specific components function as restorative stimuli. Chawla and her colleagues [35] conducted qualitative research that demonstrated that stress and hardship can lead children to seek refuge in nature for restoration and healing. The feelings, experiences and recollections reported support the previous findings of the benefits of restorative natural experiences; however their work also suggests that the restoration experience is occurring while the children are engaged in directed attention activities as opposed to the traditional belief that restoration takes place during involuntary attention activities [35]. While the underlying mechanism of restoration is debated, it has been suggested that restoration is primarily cognitive [9,36]. From the standpoint of a designer attempting to apply restoration theory in the practice of designing landscapes, producing a general restorative outcome that offers added benefit to their users, regardless of the mechanism, is the objective.

A finer scale understanding of how specific constituents in natural environments (such as trees) are restorative has not yet been teased apart, although there are strong suggestions as to the importance of trees [28]. This information is integral for designers so that they may realize the desired outcome of creating restorative landscapes. While previous research has focused on the restorative quality of green environments in general, investigations of specific elements such as trees have not yet been teased apart. There are, however, strong suggestions as to the importance of trees and they may be the most important natural feature in restorative landscapes [28]. Children growing up in contemporary urban environments often have their daily access to play and natural environments restricted to their home, school and nearby street, effectively limiting their access to restorative environments [37].

Landscape design decisions regarding which trees to plant are typically informed by ecological considerations such as the choice to use native species, practical horticultural knowledge such as plant hardiness in a given environment, overall design aesthetic principles such as balance or harmony between shapes as well as cost relative to the overall project budget. Few designers consider which tree choices may support healthy attention functioning year-round for subgroups of children being exposed to the designed environments.

#### Schoolyard Greening Case Study Setting

**Figure 3:**
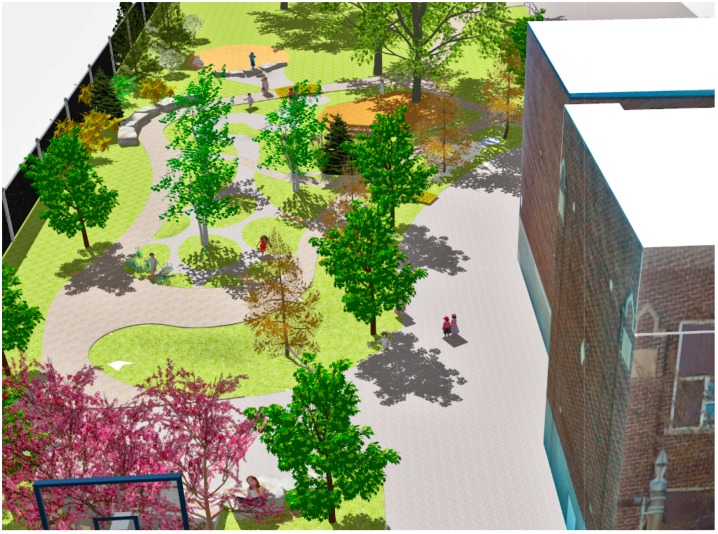
Three-dimensional model of proposed schoolyard greening intervention for visual communication and research stimuli images.

The elementary school utilized in this research as a case study site is located in an urban neighbourhood with low average household incomes and high levels of socio-economic distress in London [38]. For this study digital visualization images of a proposed schoolyard greening design were prepared using computer modeling techniques and specific research scenarios simulated for use in the production of the survey stimuli (Figure 3). The schoolyard at the case study school had a number of problems that the design intervention proposed to address. The existing conditions at the study school were perceived to pose a danger to students. Located adjacent to one of the city’s busiest streets, the case study school had been the scene of two separate traffic incidents where cars had breached the fence at the front of the school. As a result, the entire hard surfaced area at the front of the school was deemed off-limits to the children during their recess and outdoor gym periods.

While the hard surfaced areas did not offer many opportunities for outside play besides ball games and running around, this restriction nonetheless significantly limited the total area in which the children could play. More importantly, it also prevented them from accessing the small adjacent parkette that is part of their schoolyard. This area offered a variety of shade trees, some evergreens and some seating opportunities, all of which could have been beneficial to the children.

The proposed asphalt intervention sought to address these safety and usage issues so that the space could be accessible to the students while also offering some much needed garden space for play. The design also proposed to remove a large section of asphalt and replace it with natural play space that made use of trees and other plantings as restorative elements.

Based upon the design for the playground space, a three-dimensional base model of this real world greening project (not yet built at the time of this study but since completed), was created in Trimble SketchUp Pro 2013, to aid in the visual communication of the project to the public and school officials. In addition the visualizations were created to serve as the basis for rendering the stimulus images to be used in the attention restoration survey. The images represented the dynamic nature of tree foliage, specifically the changing fall colours of deciduous trees in this region of Canada, which typically includes: red, purple, orange or yellow or some variation thereof, depending upon trees species and cultivar. This phenomenon starts in late August to mid-September and extends into November. For much of the year in this zone trees are without leaves typically from late October to mid-April. That was also represented in the survey images, along with the typical green foliage of spring and summer.

## 2. Materials and Methods

### Seasonal Foliage Visualization Survey

Prior to commencing the study, ethics approval (#14-04-08-1) was obtained from the subject school, Fanshawe College and The University of Western Ontario’s Non Medical Research Ethics Boards. This study sought to test the influence of the tree planting and seasonal foliage changes by manipulating images of the proposed planting design, then presenting multiple views of various foliage conditions to the study participants. Han’s [38] Short Version Revised Restoration Scale (SRRS) is a previously validated, reliable instrument that was utilized in this research without alteration. The SRRS tool has also been utilized effectively by Han [23] with grade 8 children as respondents in a study with similar objectives. The survey was administered using projected images on an overhead projector and screen in the classroom environment with blinds drawn. Subjects, aged 9–14 in grades 4–8, responded to the survey stimulus by circling responses on a paper copy of Han’s SRRS survey. The SRRS is a multi-dimensional self-report tool comprised of eight, nine point scale questions, grouped into pairs to target four specific dimensions: (1) emotional response; (2) physiological response; (3) cognitive response; (4) behavioral response.

The SRRS showed sufficient reliability for each of the 12 images, with Chronbach’s alpha ranging between 0.80 and 0.88 (8 items). Chronbach’s alpha for the aggregated scores across the 12 images was 0.87 (8 items). The four subscales (each consisting of two items) also showed sufficient reliability. Chronbach’s alpha for the aggregated scores was 0.91 for the emotional subscale, 0.77 for the physiological subscale, 0.94 for the cognitive subscale and 0.96 for the behavioural subscale.

This research method used in this study was chosen as it builds on a well-established and commonly used methodology in environmental psychology and landscape architectural research. Traditionally the stimuli used for visual preference surveys have been photographs or photo simulations; these tools, however, have limitations in their ability to sufficiently control environmental factors in order to isolate one given element or variable [39,40]. By creating a digital model of a proposed design intervention, visualization images can be generated from several vantage points and highlighting differing environmental conditions; while the variable being investigated is manipulated, the context can be held constant, preventing or at least lessening the influence of confounding variables. To capture the responses to the various visualization images, a well established, previously validated and reliable measurement tool was used to gather projected behaviour responses to the computer generated visualizations being used as the stimuli.

The planting strategies for each foliage condition and planting strategy to be tested involved manipulating the ratio of deciduous to coniferous tree types, as well as the seasonality conditions of the trees in the images; the remainder of the scene was held constant to limit the influence of external variables. Based upon the previously described conditions, images were rendered from the digital model for use as the survey stimulus in this investigation. Each image was rated based upon Han’s SRRS to assess the perceived restoration offered by each scenario.

#### Development of Three Dimensional Model and Survey Visualization Images

Using SketchUp 2013 (Trimble, Sunnyvale, CA, USA), a model of the base design was prepared using a scaled design plan and on-site measurements of the physical space. Photos taken on site were used as context to bound the area contained in the model. The school building was modeled using the text photos taken on site SketchUp thereby allowing the use of an accurate representation of the building context. To avoid any influence from changing atmospheric conditions, a high dynamic range (HDR) image of a sky was rendered in Vue Complete 2015 (E-on Software, Beaverton, OR, USA) to provide a consistent backdrop image and lighting for all rendered scenes.

The tree components used in the model were taken from Dynascape Sketch3D (DynaSCAPE, Burlington, ON, Canada) library and these very accurate models allowed for both representation of the specific tree species in the design, as well seasonal color variations; most of the components were shown as having a fall color offering. To create a leafless condition or for those image variations where tree components were not shown with fall coloring, the components were manually edited to either remove the leaves or alter the color of the photo-based texture used to describe the leaf material.

To ensure a realistic portrayal of the scene and keep the views constant, the camera placement for all images created in *SketchUp* was set at a height of 1.6m to represent the view from vantage point of a young learner [41,42,43]. The field of view for the “camera” in *SketchUp* was set to 60 degrees to correspond with a typical field of view for a human being.

Using the case study site model, three foliage conditions were created for each of four different vantage points from around the schoolyard: (1) *Trees Inleaf with Green foliage*; (2) *Trees Inleaf with Orange foliage*; (3) *Trees Leafless*. A fourth set of images was prepared for each vantage point in order to test the impact of adding a 3:1 mix of evergreen conifers; evergreen tree components from the Sketch3D library were added in place of some of the deciduous trees present in other images, in locations that would be appropriate for the design.

The result was a set of 12 images for use in the visualization survey. The *Trees Inleaf with Green foliage* condition represents the period typical from April to September in the study region, inclusive of spring and summer. The *Trees Inleaf with Orange foliage* condition was used as a generality for the seasonal fall conditions (September to November) and was comprised of trees with color variations from yellow to orange and red. The *Trees Leafless* condition represents the period from late fall, through winter (December to March) and into early spring (March/April) in the study region (typically late October or early November) in which deciduous trees have lost their foliage or have not yet leafed out. No snow was added for the *Trees Leafless* condition as it would potentially add a confounding variable to the study and would limit the time of year, which this image could represent. Leaves were not added to the ground in the *Trees Inleaf with Orange foliage* condition images as this would have introduced a confounding variable.

Each of the three perspectives within the model was rendered as images using an internal rendering plug-in application within SketchUp called Twilight Render 2.3 (Twilight Render LLC, Castle Rock, CO, USA). The image quality was set to “High” and the image size to 1600 × 1092 pixels, which is appropriate for on-screen viewing of the visualization images. In response to feedback gathered in a previous study, the decision was made to include no people or users (entourage) in the scene to avoid any influence they may have upon the survey responses.

#### Participants

The primary researcher initially visited the subject school to introduce the study to the relevant teachers and to provide a letter of information to go home to parents to obtain parental consent. Seventy-two students (100% of eligible students) participated in the study with sixty-six (mean age 12.2) completing the survey in its entirety.

#### Survey Procedure

All seventy-two eligible students were gathered in a single room and shown the survey stimuli images via an video data projector (VDP) on the screen at the front of the room. Initially, survey participants were shown two practice images for a total of 75 s to provide them time to view the images and to read the questions on the hard copy paper survey so as to become comfortable with the procedure. The researcher, with the assistance of a colleague and the children’s respective teachers, explained the terms used in the survey, specifically the four dimensions *emotional*, *physiological*, *cognitive*, and *behavioural* and examples were provided. In preparing the students to complete the activity, emphasis was placed upon the individual questions that comprise each dimension in the survey. These individual questions use simple, easily understood terminology that was accessible to the children. Examples were also provided to illustrate the terms in each individual question. The researcher then gave an example of how to use the rating scale and the children were given the opportunity to ask questions before and throughout the activity to ensure that the children comprehended the survey. The survey images were then shown to the students for a total of 45 s each, a length of time, which has, been shown to be sufficient for measurable restorative effects to be elicited [44]. Respondents rated the images based upon Han’s SRRS to capture response to each viewed scene.

#### Perceived Restoration Scale Survey Instrument

**Figure 4:**
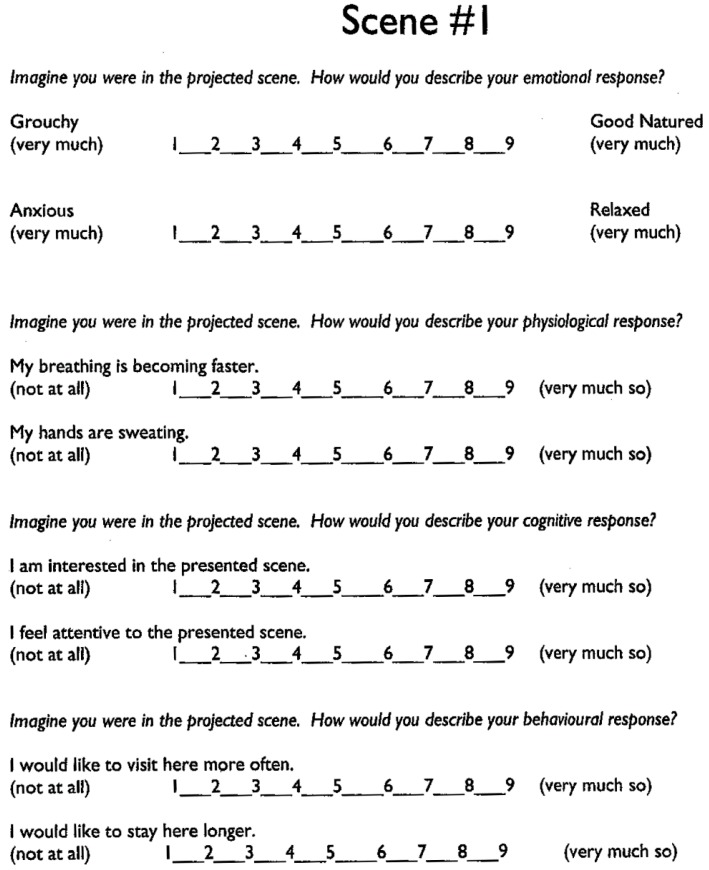
Survey questionnaire.

The survey instrument used was Han’s Short Version Revised Restoration Scale (SRRS), which is a revised version of earlier more lengthy tools created by Hartig and colleagues [13]. Hartig’s RPRS (Revised Perceived Restoration Scale) is an abbreviated version of the original Perceived Restoration Scale (PRS) [13] which measured 44 items using Kaplan and Kaplan’s (1989) ART theory focusing on mental fatigue. The PRS used short sentences in language based on Kaplan and Kaplan’s [9] theories to measure human reaction and responses to landscapes based on four dimensions: (1) extent; (2) being away; (3) soft fascination, and (4) compatibility. The PRS has been seen as too lengthy and jargon-laden; a revised tool was developed called the Revised Perceived Restoration Scale (RPRS) that uses the same 4 dimensions but with only sixteen items measured [13]. Han [38] further refined this instrument to produce a more practical, valid and reliable version in the SRRS with fewer questions, simplified language and a nine-point scale to capture the responses (Figure 4). As discussed above, identifying design solutions and the specific constituents that can provide restoration is of the most significance to the design practitioner in operationalizing theory. Han’s [38] SRRS is a tool that adopts a slightly broader notion of restoration than that in Hartig’s PRS or RPRS and, most importantly, it was designed specifically for the assessment of design and planning scenarios such as that found in the case study used in the present research.

#### Constructs and Measures

**Figure 5:**
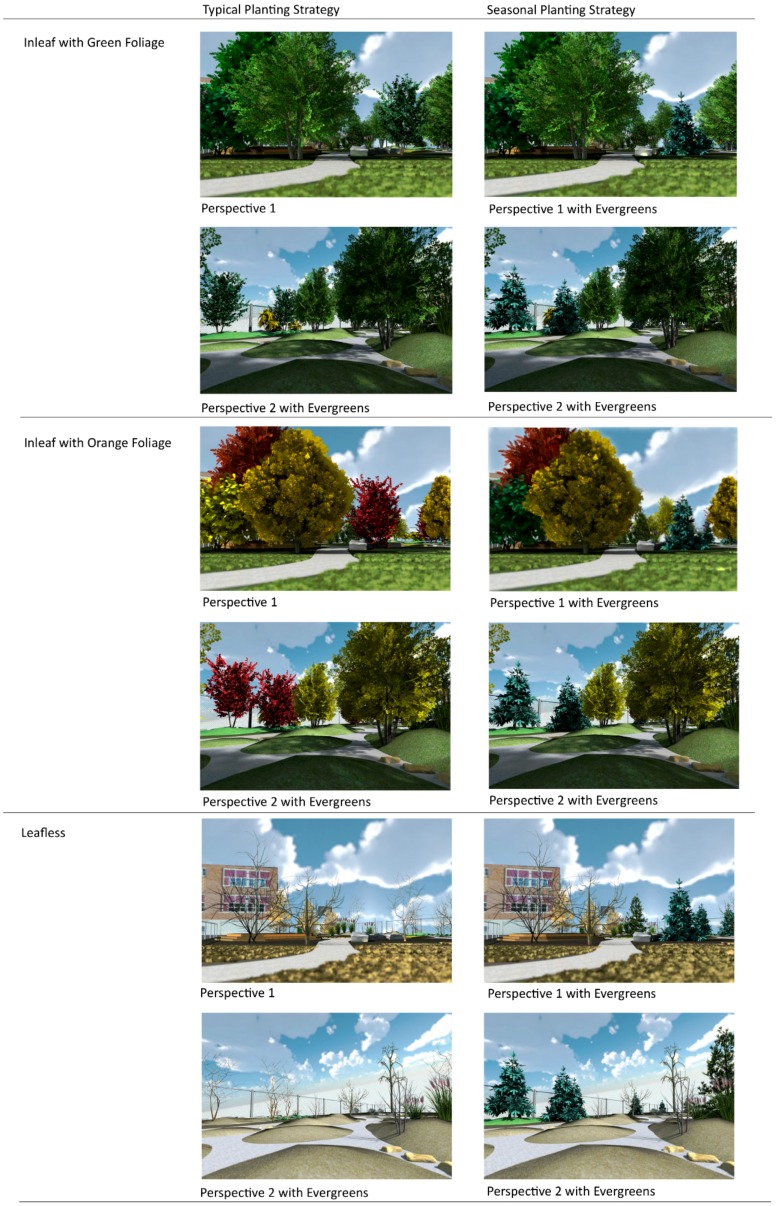
Stimulus images.

The independent variables in this research are the presence and seasonal colour of deciduous tree foliage and the introduction of evergreen conifers as a seasonal planting strategy (see Figure 5). The levels of the independent foliage variable are: *Inleaf with Green Foliage, Inleaf with Orange Foliage and Leafless.* Two images (perspectives) were used to represent each foliage condition variable. The levels of the evergreen variable were created through substituting one evergreen for every fourth deciduous tree to create a 3:1 planting ratio. Two images (perspectives) of each of the condition were created: *Inleaf with Green Foliage* *with Evergreens*, *Inleaf with Orange Foliage* *with Evergreens* and *Leafless with Evergreens*.

## 3. Results

**Figure 6:**
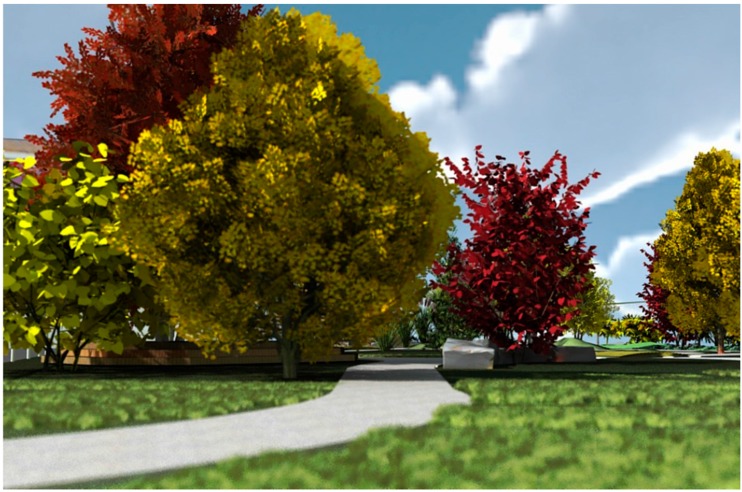
Most restorative scene in sample.

**Figure 7:**
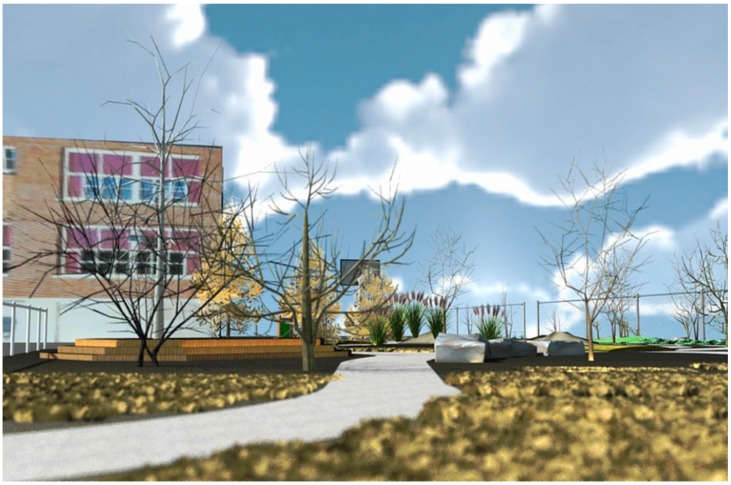
Least restorative scene in sample.

Paired two-tailed T-tests were performed on the index scores for each scene based upon the 3 conditions: (1) *Trees Inleaf with Green foliage*; (2) *Trees Inleaf with Orange foliage*; (3) *Trees Leafless* comparing the data arrays of each condition in pairwise fashion. Table 1 gives an overview of children’s mean ratings of perceived restoration and standard deviations for each of the 12 scenes. The scenes with *Inleaf* trees were generally perceived as restorative with mean values on each of the four subscales above the midpoint of the 9-point rating scale. The scenes with *Leafless* trees were generally perceived as not restorative, with means below the midpoint of the scale with the exception of the cognitive dimension. The scene that was rated as most restorative was the scene with *Inleaf* orange trees shown from perspective 1 (Figure 6). The scene rated as least restorative was a scene with leafless trees shown from perspective 1 (Figure 7).

### 3.1. Differences between Foliage Conditions

Differences in perceived restoration between the three foliage conditions (orange, green, leafless) were tested with paired *t*-tests of the average scores for each perspective with Bonferroni correction. *p* Values of less than 0.02 were considered significant. Scenes with evergreens were not included in these analyses. Scenes with *Inleaf* orange trees were rated as significantly more restorative than leafless trees, mean difference (SE) = 1.56 (±0.14), *t* = 11.40, *p* < 0.001. Scenes with *Inleaf* green trees were also rated as significantly more restorative than leafless trees, mean difference (SE) = 1.42 (±0.13), *t* = 10.58, *p* < 0.001 The difference in perceived restoration between scenes with *Inleaf* orange trees and *Inleaf* green trees was not significant, mean difference (SE) = 0.14 (±0.91), *t* = 1.57, *p* = 0.12.

### 3.2. Impact of Evergreens

**Figure 8:**
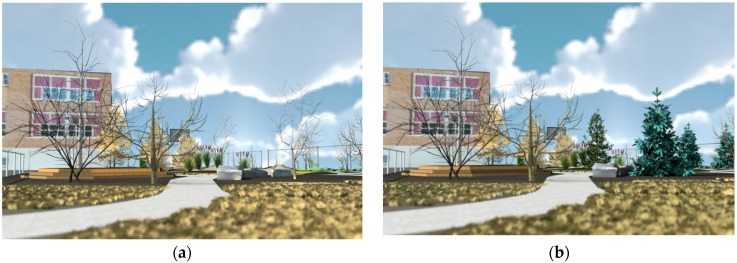
Comparison of *Leafless* condition images without (**a**) and with evergreen conifers (**b**).

Within each foliage category two of the four scenes were modified to replace some of the deciduous trees by evergreens. To test for the impact of the evergreens, the average perceived restoration scores between scenes with evergreens and scenes without evergreens were compared using paired t-tests for exploratory purposes. Results show that in general the scenes including the evergreens were not rated higher on perceived restoration than the scenes without evergreens, mean difference (SE) = 0.16 (±0.16), *t* = 1.08, *p* = 0.281. When looking at the individual impact of evergreens within the three foliage categories, there was a significant difference for the *Leafless* category (see Figure 8) and the *Inleaf with Green foliage*. The *Leafless* scene with evergreen conifers was rated significantly more restorative than the leafless scene without evergreens, mean difference (SE) = 0.90 (±0.18), *t* = 5.02, *p* < 0.001. The *Inleaf with Green foliage* with evergreen conifers was also rated significantly more restorative than the *Inleaf with Green foliage* condition without evergreen conifers, mean difference (SE) = 0.34 (±0.13). *t* = 2.65, *p* = 0.009. For the *Inleaf with Orange foliage* conditions, there was no significant difference between the leafless scene with and without evergreens, *p*-values > 0.49.

## 4. Discussion

In the present research we link theory to practice by examining different types of schoolyard designs prior to the start of a school yard greening project to produce a design that supports restoration. Children were asked to rate the perceived restorativeness of design alternatives that visualized different plantings in different seasons using the SRRS scale developed by Han [36]. The findings provide empirical support for the idea that seasonal changes in tree foliage may influence children’s perceptions of the restorative benefits of the schoolyard environment. In particular, visualizations of a schoolyard with *Leafless* trees were rated as less restorative than visualizations with *Inleaf* trees. Moreover, “orange” fall foliage was rated equally restorative as “green foliage.” The findings also indicate that the inclusion of evergreens can enhance the restorative quality of the schoolyard, especially in the winter season when trees are leafless. Taken together, this study shows that tree choice is a strategic factor in designing schoolyards that optimize year-round restorative experiences in the playground environment.

With regard to the two main research questions this study provided, the following answers can be given:

*(1)*  How do seasonal changes in deciduous tree foliage impact children’s perception of the restorative value of schoolyard trees?

This study suggests that children perceived the restoration offered by schoolyard trees as being influenced by seasonal changes in foliage. Not surprisingly, the absence of foliage that we would typically find in the study region in late fall, winter and early spring (*Trees Leafless* condition) creates an environment that was not perceived by participants as being very restorative. With the understanding that children spend approximately half of the school year in conditions of this nature, it seems very likely that this condition impacts upon their attention functioning and academic performance in the classroom during those seasons. When considered in the existing context of previous studies such as Faber Taylor, Kuo and Sullivan’s [25] study of children with ADD and the importance of *green* playground spaces, this research both agrees with their findings of the attention benefits provided by these exposures, while at the same time suggesting that further fine tuning may be necessary so that these benefits can continue to be received as tree foliage changes in colour or disappears according to the season. Students with attention function disabilities, such as ADD or Attention Deficit Hyperactivity Disorder (ADHD), may find their ability to mitigate the condition through the mental restoration that would otherwise be provided during recess in those seasons when the trees have foliage, lessened in those seasons where trees are without leaves.

Surprisingly the *Trees Inleaf Orange Foliage* condition was rated as providing equal levels of perceived restoration as the *Trees Inleaf Green Foliage* condition. The potential negative associations attached to the fall season, as the harbinger of winter, were expected to negatively influence the response to the fall colour scenes, but that does not appear to be the case. As most attention restoration studies focus on “green” environments as the restorative binary opposite to urban environments, we may have to rethink this relationship, as it appears that perhaps “orange”(or red or yellow) is at least as restorative as “green” when it comes to foliage. In fact the rankings showed two of the fall foliage conditions (*Trees Inleaf Orange Foliage)*, were rated the highest in the sample, which suggests that perhaps further investigation of fall foliage colour may be warranted.

*(2)*  Does the addition of conifer trees extend the restorative effect of schoolyard plantings during times when deciduous trees have no foliage?

Student participants perceived the use of a seasonal planting approach, that includes evergreen trees, as having greater restorative effect in the *Trees Leafless* scenarios that would be representative of late fall, winter and early spring. Although the ratings were the lowest overall for all of the *Trees Leafless* conditions, when conifers were added to each of these scenes, they were rated as having greater perceived restoration than scenes where deciduous trees had no visible leaves. This is a very important finding as it validates a long held belief among designers, that seasonal interest in planting design leads to better landscapes year round. Now we may have signs that point to potential reasons as to why.

Beyond providing aesthetic appeal, seasonal plantings that include evergreens may serve to enhance the restorativeness of the landscape. It is further suggested that even in those seasons with an abundance of green foliage (spring or summer), the introduction of evergreen conifers may increase the restorative quality of the landscape. While the change in perceived attention index scores was small overall when comparing the *Trees Leafless* scenes with and without evergreens, the lived experience produces a more pronounced effect and should be tested through further research. Han’s [23] study of the influence of including plants in children’s classroom showed positive influence upon both perceived health and a reduction in reported behavioral incidents and absences due to illness, which indicates that small interventions as part of children’s school day experience may provide significant benefits. Adding evergreens to the school playgrounds of children living in regions where trees are predominantly deciduous may provide a small improvement in perceived restoration, as suggested in this study. There is also potential for there to be other healthful benefits from seasonal planting strategies that may aid in combating seasonal health conditions, from flu to seasonal affective disorder, to which children may be subject in northern climates.

This research adds to a growing body of research on children’s environment and behavior from disciplines of Geography, Environmental Psychology and Landscape Architecture that suggest that natural environmental exposure, in this case specifically to trees, are perceived to be healthy components in children’s learning environments. What is novel about this work is that the results suggest that the differences in seasonal variation in deciduous tree foliage creates a corresponding variation in the healthful attention functioning benefits provided by this environmental exposure.

This study supports some long standing assumptions and practices in the landscape design field regarding the importance of planting for seasonal interest. Having evidence to support design decisions in schoolyard environments is of great importance as the process of making changes to schoolyards is often a laborious and bureaucratic process requiring many levels of approvals in order to realize projects with very limited budgets to fund them. This research suggests the need to make decisions that maximize the impact of small budgets to produce the most supportive environments for children.

As expected, the lack of foliage in the late fall, winter and early spring, creates an environment that is perceived as having low restorative value for the school children that would experience it. As one would imagine, providing a landscape that supports attention functioning in the cold Canadian winter landscape, when deciduous trees are leafless, is a challenge. This study demonstrates that there is a significant difference in the perceived restoration of the *Trees Leafless* condition if evergreen conifers are added to the planting mix. Landscape design professionals have attempted to combat the lack of “green” in the leafless periods through planting evergreens for seasonal interest in many other contexts, but rarely is this done in school greening projects. The focus of schoolyard greening tends to be upon the provision of shade, which is not a feature offered by most evergreens in the region studied; however this study shows that there is a functional justification for their inclusion. Evergreens improve the perceived restorativeness ratings in elementary school children and therefore this design approach is expected to support healthy attention functioning in the months with little foliage offered by deciduous tree types. Another practical consideration is the lower cost of evergreen conifers *versus* deciduous trees, which has significance in the context of the limited budgets that typically constrain schoolyard greening projects. Given the length of time that trees are in the leafless condition during the school year in most Canadian cities, up to half of the school year (four to five months), design interventions that improve the low restorative capacity of the schoolyard are very important, especially in those schools where socio-economic distress levels are high and the need for attention restoration is likely in greater demand.

Perhaps the most interesting finding of this study is that the scenes representing fall colors were equally or even somewhat higher in their levels of perceived restoration offered than green scenes. Previous attention restoration studies have predominantly focused on scenes of green environments without consideration of seasonal change. While this period of brilliant color only lasts for a few weeks each year (just after the school year begins in most Canadian cities), there is potential to extend this impact through informed plant choices and perhaps to enhance the restorative quality of the foliage in the remaining portions of the year. Some tree species and cultivars offer foliage color that is similar to that found in the fall season or else offer purple leaf variants that are common to the residential landscape but not typically used in school yard designs. Both of these options may add a fall-like color to the predominantly green palette of spring and summer thereby enhancing their restorative capacity. Strategically choosing deciduous plantings based upon when they produce fall color so as to extend the seasonal foliage color may also help to maximize the restorative value of the schoolyard landscape. Although flowering ornamentals were not explored in this study (as they are typically avoided in school yard design) these plantings may also offer higher restorative values and should be investigated in future research.

For school administrators, landscape design professionals and the groups that work to improve the quality of schoolyard landscapes, the choices of which trees to plant and where are decisions of great importance with long term impact. Frequently, with limited resources, tree planting is limited in number, therefore achieving the maximum benefit for the student users is of the utmost importance and this study has provided some valuable information to aid in making functional choices that provide support for the healthful attention functioning for children.

On a methodological level this study demonstrates the utility of using computer generated visualization images as a means to isolate environmental components for study to limit the influence of confounding variables, and thereby addresses one of the major criticisms of image-based environmental investigations. As a tool for the generation of experimental stimuli, simple computer modeling and visualization were shown to be an innovative and highly effective means of exploring environmental issues that are otherwise difficult to assess.

There are of course, some limitations to this study. Han’s SRRS has not been used with children as young as the sample in this group and there is no established reliability or measure for this specific age group. Han [23] surveyed grade 8 children, with a mean age of 13.6 and the mean age of the children in the research presented here was 12.2 years, however the SRRS tool used was developed using college students (average age 19 years) [38]. It is acknowledged that there is a validated restoration scale tool for use with children (PRCS-C), however this tool is lengthy and not as well suited to practical design and planning scenarios as Han’s SRRS [45].

The number of scenes representing each condition was also small in this research so there is the potential for mono operation bias in this study. Not representing a snow condition is another limitation of the study, as it fails to address a condition that is typical for several months of most school years in the case study region; however, the snow would have introduced a confounding variable to the study, thereby making it difficult to examine trees specifically. Fallen leaves were also not added to the condition representing the fall season, as this was believed to also offer the potential of introducing unwanted outside variables. Further research should explore the influence of snow and other meteorological conditions on restoration.

While imaging, in this instance computer generated imaging, is a widely used surrogate for a real world experience, the fact that it is not a real world exposure is a limitation of this research model. A weakness of this approach is that the response is subjective in nature and projected rather than measuring objective physiological responses to a real world exposure; however there has been considerable research comparing this method to real world objective approaches and the findings indicate that the methods produce results that are in accordance with one another [39,40,46,47]. In this study, we asked children to imagine themselves in their own schoolyard, in a designed space that they have participated in creating through a participatory design process that preceded this research. This is a space that they were very familiar with and have experience mentally reconfiguring as part of the design activity. Image based studies do tend to pose the question of whether the research participants are responding to the scene visualized or just the image itself; however given that this was their own schoolyard, it seems unlikely that this group of respondents would not be evaluating actual space.

Cultural and psychological associations in response to the colour change in foliage or the absence of foliage could potentially also have an influence upon the perceived restoration ratings in an unanticipated manner therefore this is another limitation of this study. The Leafless condition does present a much more open landscape which could trigger responses relating to that perceived change which could induce a stress response thereby reduce perceived restoration.

## 5. Conclusions

**Figure 9:**
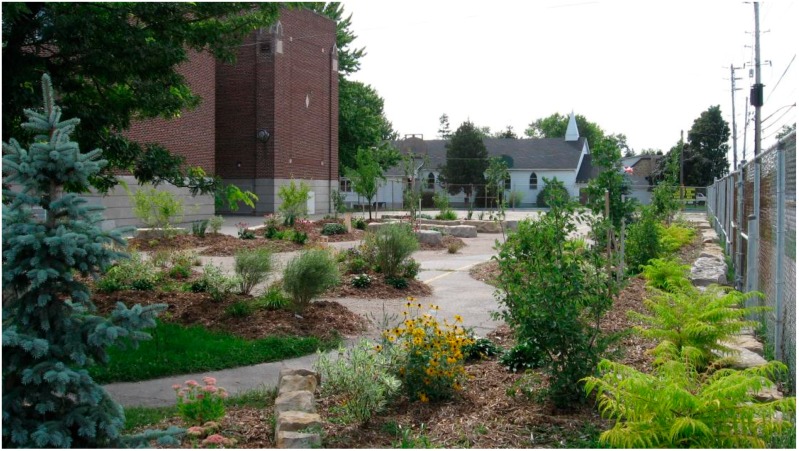
Subject schoolyard “greened” with seasonal planting strategy implemented.

Ultimately the findings of this research and their value to real world design will depend upon how well the results match up with the actual experience of restoration (not just self reports of projected experiences) in response to experience of the actual environment (not just visualizations of proposed design scenario). The design was installed in the spring of 2015 and is heavily used by the children who participated in the research (see Figure 9) so there exists a great opportunity for further research to expand on the findings and address some of the limitations discussed above.

## Figures and Tables

**Table 1:** Means and standard deviation (in parenthesis) for subscale scores and overall SRRS score.

Scene	Emotional	Physiological	Cognitive	Behavioural	Overall
*Inleaf with Green Foliage*					
Perspective 1	6.05(2.46)	5.41(2.46)	7.59(2.49)	5.03(2.52)	5.97(1.71)
Perspective 2	6.05(2.65)	5.47(2.60)	7.33(2.65)	5.68(2.73)	6.08(1.83)
Perspective 1 with Evergreens	6.58(2.45)	6.93(2.55)	7.71(2.08)	5.82(2.72)	6.33(1.69)
Perspective 2 with Evergreens	6.52(2.34)	5.68(2.46)	7.70(2.10)	5.42(2.67)	6.39(1.81)
*Inleaf with Orange Foliage*					
Perspective 1	6.80(2.35)	5.98(2.68)	7.38(2.50)	5.98(2.69)	6.54(1.92)
Perspective 2	6.56(2.68)	5.67(2.88)	7.20(2.80)	5.53(3.00)	6.24(1.97)
Perspective 1 with Evergreens	6.51(2.31)	5.87(2.60)	8.03(1.95)	5.61(2.61)	6.41(1.64)
Perspective 2 with Evergreens	6.34(2.45)	5.73(2.62)	7.40(2.62)	5.47(2.84)	6.20(1.88)
*Leafless*					
Perspective 1	3.72(2.59)	3.02(2.42)	7.39(2.34)	2.78(2.32)	4.28(1.81)
Perspective 2	3.74(2.51)	3.33(2.33)	7.21(2.68)	2.97(2.31)	4.34(1.66)
Perspective 1 with Evergreens	5.77(2.44)	4.83(2.62)	7.45(2.16)	4.97(2.77)	5.68(1.76)
Perspective 2 with Evergreens	4.71(2.32)	3.71(2.23)	7.30(2.31)	3.58(2.28)	4.84(1.55)
